# Silicon changes C:N:P stoichiometry of sugarcane and its consequences for photosynthesis, biomass partitioning and plant growth

**DOI:** 10.1038/s41598-020-69310-6

**Published:** 2020-07-27

**Authors:** Joaquim José Frazão, Renato de Mello Prado, Jonas Pereira de Souza Júnior, Davi Rodrigo Rossatto

**Affiliations:** School of Agricultural and Veterinarian Sciences, São Paulo State University, Jaboticabal, São Paulo 14884-900 Brazil

**Keywords:** Plant physiology, Plant sciences, Environmental sciences, Chemistry

## Abstract

Silicon (Si) application has improved yield and stress tolerance in sugarcane crops. In this respect, C:N:P stoichiometry makes it possible to identify flows and interaction between elements in plants and their relationship with growth. However, few studies have investigated the influence of Si on physiological variables and C:N:P stoichiometry in sugarcane. As such, this study aimed to assess the effect of increasing Si concentrations on the growth and stoichiometric composition of sugarcane plants in the early growth stage. The experiment was conducted in pots, using four Si concentrations (0, 0.8, 1.6 and 3.2 mM). Biomass production, the concentration and accumulation of C, N, P and Si as well as the relationship between them were assessed. Silicon application increased biomass production, the rate of photosynthesis, instantaneous carboxylation efficiency and C, N, P and Si accumulation, in addition to altering stoichiometric ratios (C:N, C:P, N:P and C:Si) in different parts of the plants. The decline in C concentration associated with greater N and P absorption indicates that Si favoured physiological processes, which is reflected in biomass production. Our results demonstrate that Si supply improved carbon use efficiency, directly influencing sugarcane yield as well as C and nutrient cycling.

## Introduction

Silicon (Si) is the second most abundant element in the earth’s crust^1^. However, a significant portion of Si is not readily available to plants, particularly in highly weathered soils such as those found in the tropics, where most global crop production is concentrated. Although Si is not considered a nutrient, in plants such as sugarcane, a known silicon accumulator^2^, its presence favours growth and biomass production^3–7^.

The processes responsible for the high growth rate observed in sugarcane and crops such as rice, pea, maize and wheat^8,9^ supplied with Si include mitigating different stresses^10,11^, especially those that affect photosynthetic capacity, and improving physiological processes due to nutrient accumulation and use^12,13^.

Photosynthetic capacity is generally closely linked to leaf N and P content^14–16^. Previous studies have shown that N and P deficiencies reduce the photosynthetic rate (A), stomatal conductance (gs), transpiration (E) and chlorophyll content of plants^15,17–19^. However, some studies have suggested that elements such as Si may affect photosynthetic performance, even in plants grown under stress^20^. Silicon has been reported to increase A and chlorophyll content^9,21,22^, with significant impacts on leaf N and P content^9^.

On the other hand, recent studies have revealed that Si can reduce carbon (C) concentration in wheat plants^23^, suggesting that it may play a role similar to C in plant leaf structure. In fact, there is evidence that the energy cost of including Si in the C chain is lower than that of C inclusion^24^. As a result, increasing doses of Si can alter the stoichiometric composition of plants and, in turn, improve their physiological aspects, leading to increased growth. Recent investigations have found that Si application can change the stoichiometric ratios in wheat^23^ and rice leaves^25,26^.

Although sugarcane is one of the world’s most widely grown crops^27^ and a well-known Si accumulator, there are no studies that investigate the effect of Si on C, N, and P accumulation, their stoichiometric ratios and physiological variables such as A. The relationship between Si and stoichiometric homeostasis and its impact on gas exchange needs to be better understood in this species, which is known to have highly efficient biomass accumulation.

The aim of this study was to determine whether there is a dose-dependent response to exogenous Si application in the C:N:P stoichiometry, physiological variables and growth of sugarcane plants. We hypothesized that an increase in Si supply will: (1) modify C:N:P stoichiometry, in particular by decreasing C content without affecting N and P, and (2) improve photosynthetic parameters, leading to better biomass production in sugarcane plants.

## Results

### Plant growth

**Figure 1:**
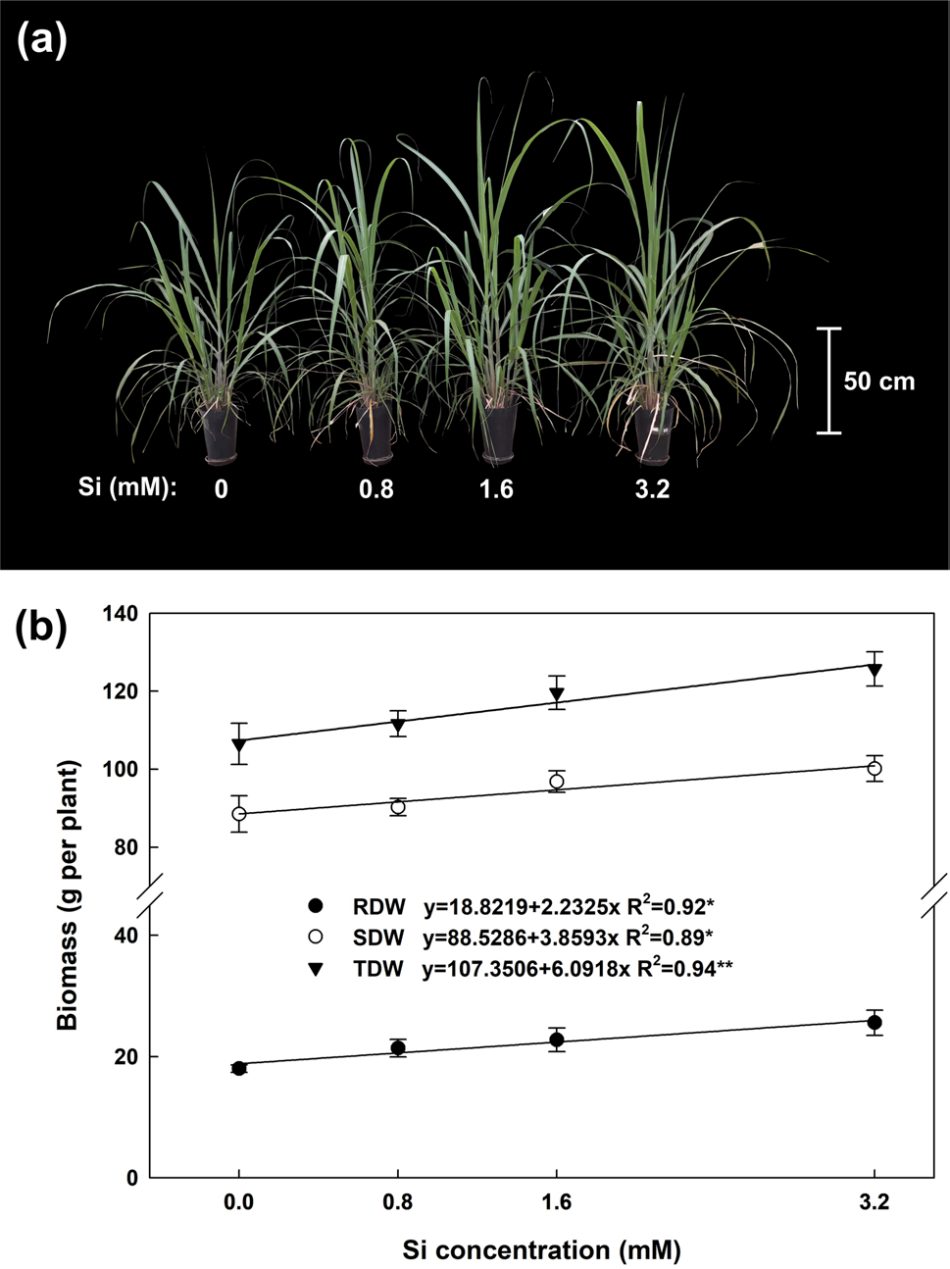
(**a**) Response of sugarcane plants supplied with increasing concentrations of silicon at 90 days after transplanting. (**b**) Shoot dry weight (SDW), root dry weight (RDW) e total dry weight (TDW) of sugarcane plants supplied with increasing concentrations of silicon. Error bars indicate standard error of the mean (n = 6).

Silicon application caused a linear increase in shoot (SDW) and root dry weight (RDW) and, consequently, the total dry weight (TDW) of sugarcane plants (Fig. 1). SDW contributed to approximately 80% of TDW, which varied from 106.5 to 125.7 g per plant.

Silicon application increased TDW by up to 18% (4.8–18%) in relation to the control treatment (no Si applied). This increase was even more significant on analysis of RDW data, where the mean for controls was up to 41.8% lower than in Si treatments (18.6–41.8%). On average, Si application raised RDW, SDW and TDW production by 28.9, 8.2 and 11.7%, respectively.

### Photosynthetic parameters

**Figure 2:**
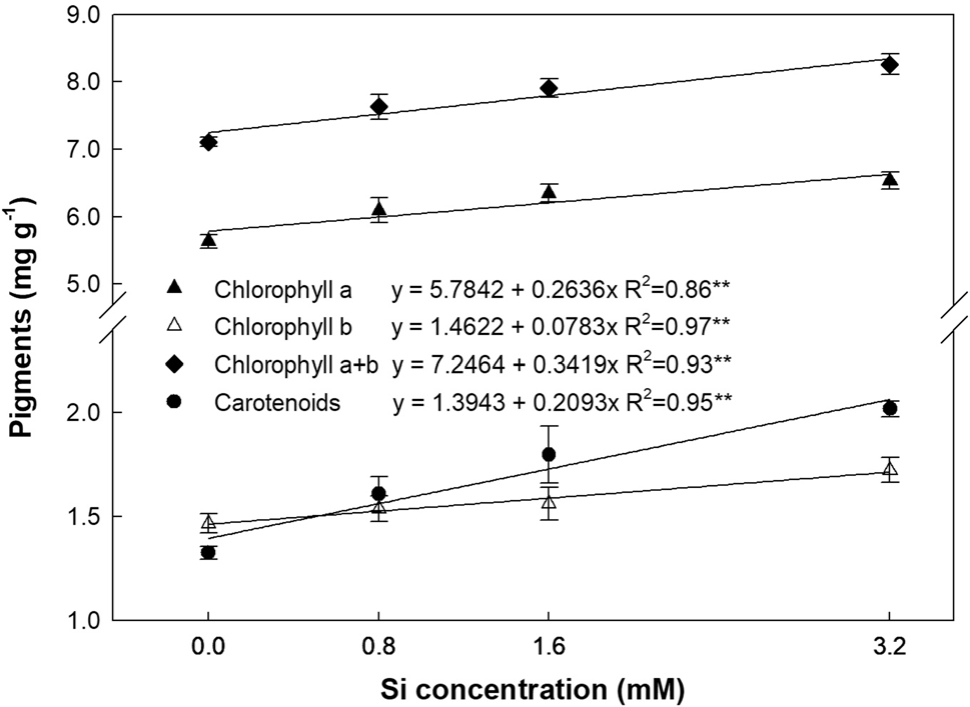
Content of carotenoids, chlorophyll a, b and total (a + b) in leaves of sugarcane plants supplied with increasing concentrations of silicon. Error bars indicate standard error of the mean (n = 6).

Chlorophyll a, b, total chlorophyll (a + b) and carotenoid content rose linearly (*P* < 0.01) with increasing Si concentrations (Fig. 2). The highest increase was recorded for carotenoid content, reaching up to 52.3% when compared to controls, with increases of 15.9, 17.5 and 16.2% for chlorophyll a, b and total chlorophyll, respectively. Carotenoids accounted for 17.8% of the total pigment content and total chlorophyll (a + b) 82.2%; 79.9 and 20.3% of which corresponded to chlorophyll a and b, respectively.

**Figure 3:**
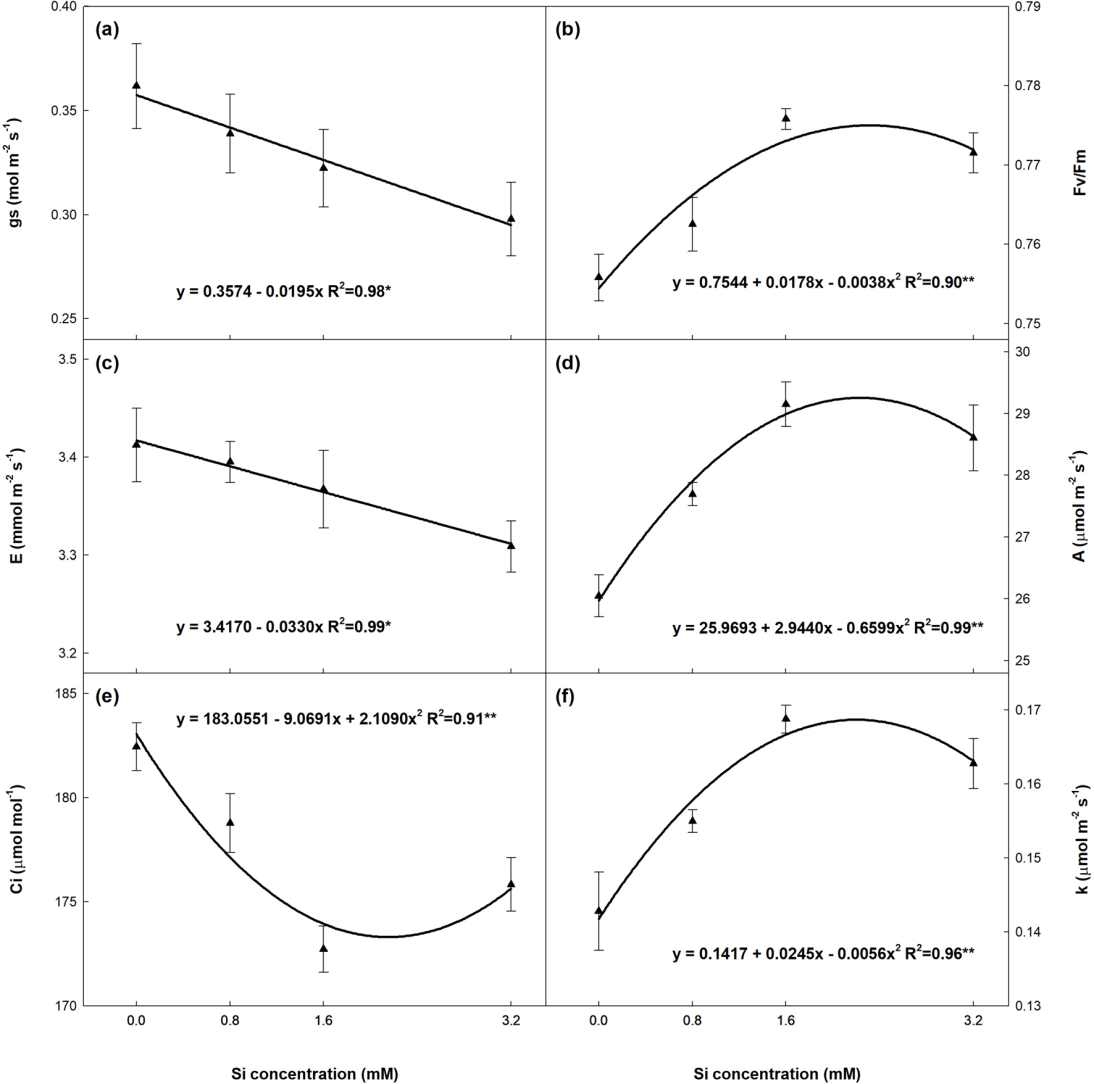
(**a**) Stomatal conductance (gs), (**b**) quantum efficiency of photossystem II (Fv/Fm), (**c**) transpiration (E), (**d**) net photosynthesis rate (A), (**e**) intracellular CO_2_ concentration (Ci) and (**f**) instantaneous carboxylation efficiency (k = A/Ci) in leaves of sugarcane plants supplied with increasing concentrations of silicon. Error bars indicate standard error of the mean (n = 6).

All the photosynthetic gas exchange parameters were significantly influenced by Si supply (Fig. 3). There was a linear decrease in stomatal conductance (gs) and transpiration (E) as Si concentrations rose and a quadratic decline for intracellular CO_2_ concentration (Ci), but a significant increase in net photosynthesis rate (A) and instantaneous carboxylation efficiency (*k*). A similar trend was observed for the maximum quantum efficiency of photosystem II (Fv/Fm). Based on the regression coefficients, the estimated maximum A (29.25 µmol m^-2^ s^-1^) and minimum Ci (173.31 µmol mol^-1^) were obtained with the application of 2.23 and 2.15 mM of Si, respectively.

### Concentration and accumulation of C, N, P and Si in plant tissues

**Figure 4:**
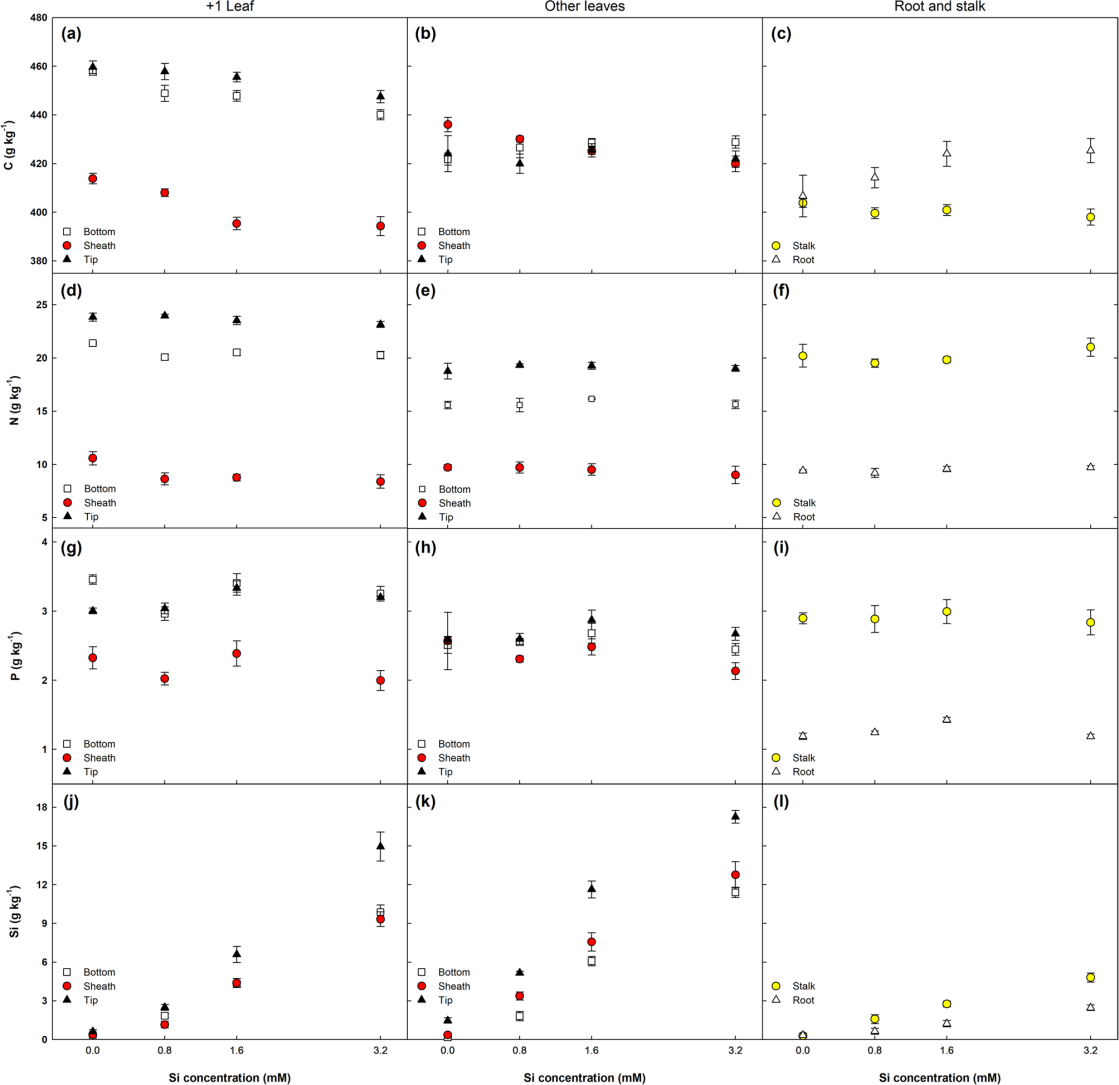
Concentration of carbon (C), nitrogen (N), phosphorus (P) and silicon (Si) in different parts of sugarcane plants supplied with increasing concentrations of Si. Error bars indicate standard error of the mean (n = 6).

Silicon application affected carbon (C), nitrogen (N), phosphorous (P) and silicon (Si) concentration differently in different parts of the plant (Fig. 4). In general, the highest concentrations were recorded in leaf + 1, followed by other leaves (OL), stalks and roots.

Silicon supply significantly altered C concentration in leaf + 1 (+ 1T, + 1B e + 1S), OL and roots, with linear declines in C content as Si concentration increased (Fig. 4a–c). The opposite occurred in roots, with a slight decrease in C concentration in the remaining plant parts.

**Figure 5:**
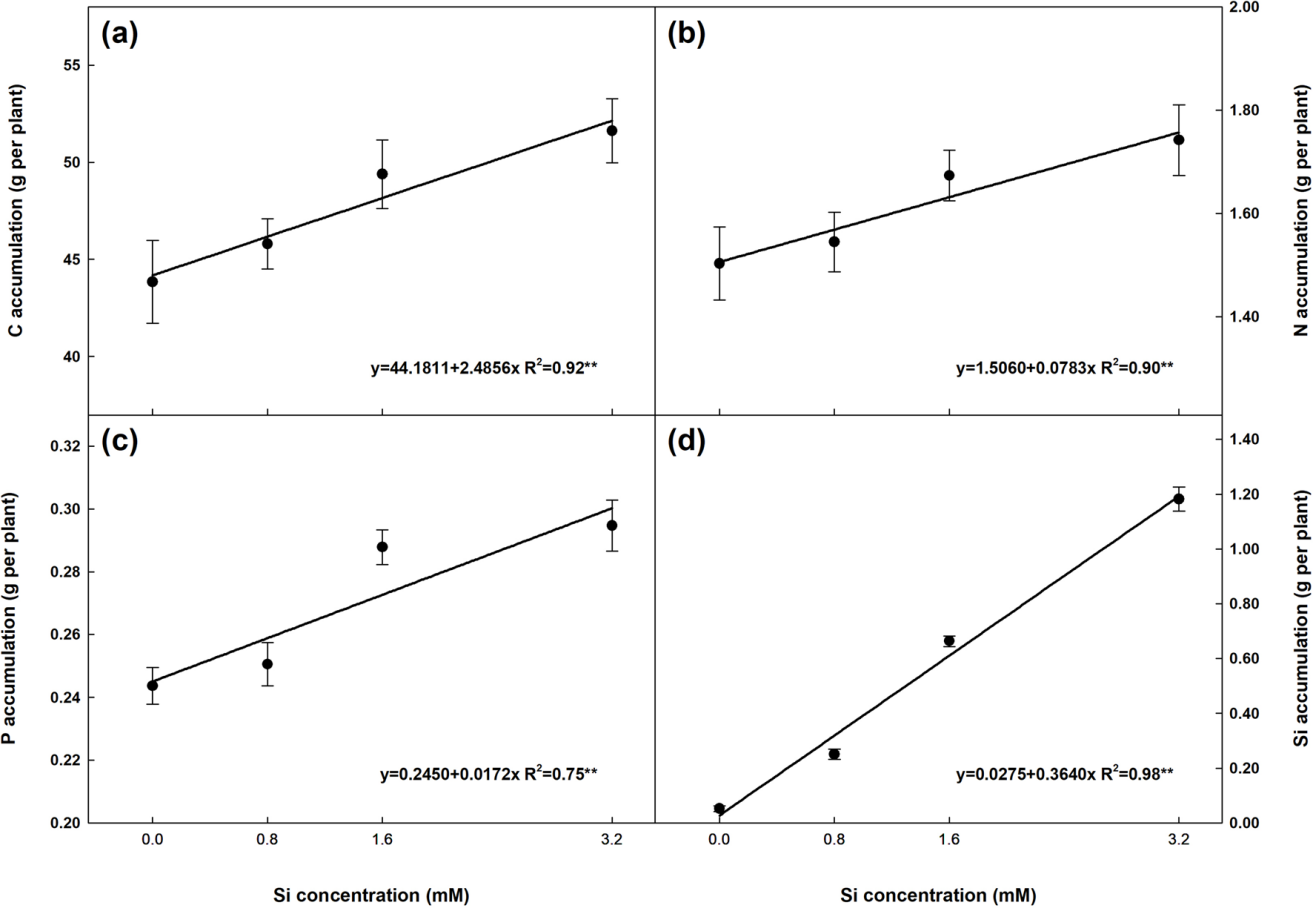
Accumulation of carbon (C), nitrogen (N), phosphorus (P) and silicon (Si) in sugarcane plants supplied with increasing concentrations of Si. Error bars indicate standard error of the mean (n = 6).

In regard to N, Si only had an effect on leaf + 1 (+ 1B e + 1S), with linear declines as Si concentrations rose (Fig. 4d). Silicon only influenced P concentration in the leaf blade (+ 1T e + 1B, Fig. 4g) and roots (Fig. 4i). Finally, Si concentration in all plant parts increased linearly (*P* < 0.01) with a rise in Si doses. As expected, Si concentration in all three leaf parts (+ 1 and OL) was considerably higher than in stalks and roots. Accumulation of C, N, P and Si in the plant increased linearly (*P* < 0.01) with Si concentrations by up to 17.7, 15.9 and 20.9%, respectively (Fig. 5). The largest variation was recorded for Si accumulation (Fig. 5d), with an average of 0.05 g per plant in controls, whereas plants supplied with Si exhibited accumulation of up to 1.18 g per plant (3.2 mM Si), representing a 2,182.8% increase.

### Stoichiometry

**Figure 6:**
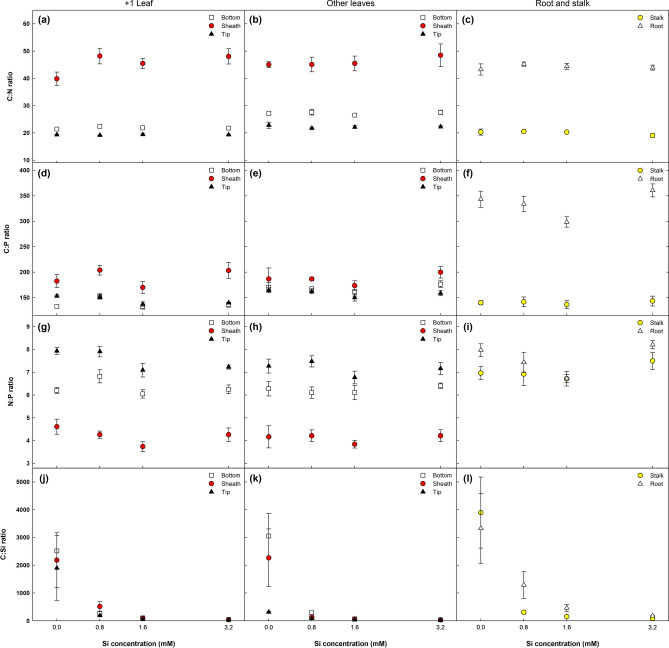
Changes in C:N:P stoichiometry in different parts of sugarcane plants supplied with increasing concentrations of silicon. Error bars indicate standard error of the mean (n = 6).

The application of Si significantly altered all the stoichiometric ratios (C:P, N:P e C:Si), except for C:N (Fig. 6). The highest stoichiometric changes were recorded for roots and leaf + 1, particularly the tip and base of leaf blades (+ 1T and + 1B, respectively). The greatest difference observed among plant parts was between the leaves (+ 1 and OL) and other parts (stalks and roots).

Stoichiometric ratios differed between OL and leaf + 1, with the former exhibiting a 15.2, 24.5 and 1.4% higher C:N ratio than the latter in the tip, base and sheath, respectively, while the C:P ratio was 9.3, 22.2 and 1.7% greater than in leaf + 1 (Fig. 6). The opposite occurred for N:P and C:Si ratios: + 1T, + 1B and + 1S were 5.2, 1.7 and 2.6% (N:P), and 381.8, 18.4 and 13.8% (C:Si) higher than OLT, OLB and OLS, respectively.

Although the Si doses used had no effect on the C:N ratio, differences were observed between the various plant parts (Fig. 6a–c). With respect to leaves (+ 1 and OL), the C:N ratio of the sheath (+ 1S and OLS) was considerably higher (102%) than at the tip (+ 1T and OLT) and base (+ 1B and OLB). The base of the leaf blade exhibited a slightly higher C:N ratio than at the tip in both leaf + 1 and OL, with a similar ratio in the sheath and roots. There was little variation in the C:N ratio in stalks (19.1 a 20.5) and roots (43.2 a 45.5) as Si concentrations increased. In descending order, the highest average C:N values per plant part were OLS >  + 1S > root > OLB > OLT >  + 1B > stalk >  + 1T.

Silicon concentrations only influenced the C:P ratio in leaf + 1 blades (+ 1T e + 1B) and roots, with the highest and lowest values recorded in the roots and stalk, respectively (Fig. 6d–f). Regardless of the type of leaf, the sheath (+ 1S and OLS) displayed a higher C:P ratio that the other leaf parts. In descending order, the largest average C:P values per plant part were root >  + 1S > OLS > OLB > OLT >  + 1T > stalk >  + 1B.

In regard to the N:P ratio, Si only had a significant effect on the roots and tip of leaf + 1 (+ 1T), with linear and quadratic adjustments, respectively (Fig. 6g–i). Patterns for the N:P ratio differed between leaf parts for both + 1 and OL: + 1T/OLT >  + 1B/OLB >  + 1S/OLS. In descending order, the highest average N:P values per plant part were roots >  + 1T > OLT > stalk >  + 1B > OLB >  + 1S > OLS.

As expected, C:Si was the stoichiometric ratio most affected by Si supply, with significant declines observed in all the plant parts, the largest from 0 to 0.8 mM of Si (Fig. 6j–l). In descending order, the highest average C:Si ratios per plant part were roots > stalk > OLB >  + 1B >  + 1S > OLS >  + 1T > OLT.

## Discussion

### Plant growth and accumulation of C, N, P and Si

Although not considered a nutrient, Si has had proven beneficial effects on the growth and production of sugarcane plants, since in a recent study (with two sugarcane varieties), yield increased linearly with Si doses^6^. Our findings confirm this beneficial effect, since linear increases (*P* < 0.01) were observed in biomass production (Fig. 1); however, we also demonstrate that these increases are likely due to the boost in photosynthetic activity in treated plants, combined with better water use and higher protection against oxidative stresses in their photosynthetic apparatus.

**Figure 7:**
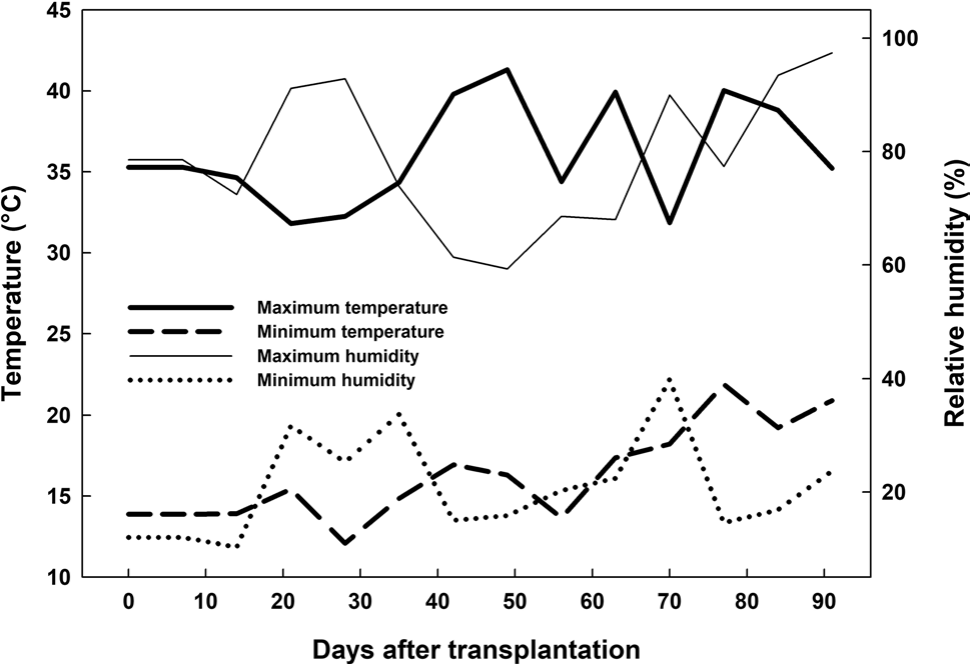
Temperature and relative air humidity during the experiment.

As a silicon accumulating species, sugarcane is highly responsive to Si application; however, another factor that contributed to these results was the climate conditions during the experiment. The optimum temperature for early sugarcane growth is between 28 and 30°C^28^, but temperatures during the study period were over 41 °C or below 17 °C, with a daily variation of up to 25 °C (Fig. 7), characterising abiotic stress.

Abiotic stress increases the production of reactive oxygen species (ROS) (O_2_^-^, OH^-^ and H_2_O_2_), which causes oxidative damage at cellular level and degrades photosynthetic pigments, hampering plant growth. In this context, Si application can mitigate the harmful effects of oxidative stress in the photosynthetic apparatus, which was in fact observed in the sugarcane plants studied, since the higher Si doses increased the leaf Fv/Fm ratio (Fig. 2), indicating reduced stress. In a recent review, Kim et al.^11^ found that Si increased the activity of antioxidants such as carotenoids, which eliminate ROS from cells.

Mitigating oxidative stress preserves the photosynthetic pigments and maintains the quality and rate of photosynthesis^29^. The larger Fv/Fm values obtained under high Si doses may be due to an increase in the concentration of pigments such as carotenoids, chlorophyll a and b and total chlorophyll (Fig. 2), as reported in recent studies that confirmed the role of Si in improving Fv/Fm under abiotic stress^13,30,31^.

In addition to preserving photosynthetic pigments, Si also improves the efficiency of the photosynthetic apparatus, largely because Fv/Fm values are related to an increase in the electron transport rate, which may result in greater carbon fixation during the Calvin-Benson cycle^32^. Another important aspect is the decline in stomatal conductance (gs) and improved water use efficiency, which help maintain the plant hydrated and increase photosynthetic activity^10^. With lower gs (Fig. 3a), there was a significant increase in net photosynthesis (A, Fig. 3d) and instantaneous carboxylation efficiency (*k*, Fig. 3f). The decline in Ci is related to the rise in A, since more electrons are available for carbon fixation. Other authors have also reported an increase in the photosynthetic rate of sugarcane submitted to Si application^21,33^, but did not correlate it with a rise in photoprotective pigments. Thus, our hypothesis (ii) was confirmed, given that the improvement in photosynthetic parameters with a rise in Si doses may be responsible for increased biomass production.

Higher biomass production (Fig. 1) combined with changes in C, N, P and Si concentrations in the different plant parts (Fig. 4) led to a linear accumulation of these elements in the plant (Fig. 5). The present study demonstrated that although Si application had no significant effect on C, N, and P concentration in some plant parts, accumulation of these elements in the plant was significant, indicating that Si also improves their absorption, as reported in previous studies^8,9,23,34^.

As expected, the highest proportional increase recorded was for Si accumulation (Fig. 5d). This is because sugarcane is a known Si accumulator and ultrapure water was used to prepare the nutrient solutions, which resulted in a greater Si accumulation gradient between controls and the highest Si concentration (2,182.8% increase).

### Effect of Si on stoichiometry

As expected, the rising Si doses influenced the stoichiometric ratios in different plant parts (i). Our findings indicate that increasing the Si supply reduced C concentrations in most plant parts (Fig. 4), representing a negative correlation between Si doses and C. This trend has also been reported in prior research^23,26,35^. The reduced C content in plant tissue may indicate its substitution by Si to form cell wall components^23,36^, contributing to better plant growth. On the other hand, the improvement in photosynthesis (Fig. 3) prompted a linear increase in C accumulation in the plant (Fig. 5a), confirming the findings of a recent meta-analysis^37^.

The slight variation in the C:N ratio in the different tissues (Fig. 6) is due to the decline in N and C concentrations with the rise in Si doses (Fig. 4). However, the increase in C:N was more marked in the sheath of leaf + 1 and OL (+ 1S e OLS, respectively) because the C concentration in these tissues declined most significantly, whereas levels in the roots rose with Si application, showing a positive correlation with RDW (Fig. 1). Nevertheless, there was no clear trend in C:N ratio alterations in this tissue. This pattern of decreased C:N ratios with rising Si concentrations may be related to the decline in lignin content, as previously reported^9,36^.

The different parts of leaf + 1 (+ 1T, + 1B e + 1S) exhibited higher N and P concentrations than those of OL (OLT, OLB e OLS), due to the redistribution of N and P from older tissue to the meristem, resulting in lower levels than in younger leaves (leaf + 1). Additionally, P and particularly N transfer to the meristem is directly linked to transpiration (E), which was negatively related to Si application (Fig. 3c), leading to a decline in C:N, C:P and N:P ratios with increasing Si concentrations. This also partially explains the slight rise in these ratios in OL when compared to leaf + 1.

There was no clear trend for the influence of Si doses on altering C:P and N:P in the different plant parts (Fig. 6d–f), although an effect was observed in the roots and leaf + 1 blades (+ 1T e + 1B). In general, C:P and N:P ratios rose with an increase in Si dose from 0 to 0.8 mM, declining from 1.6 mM Si and rising again at the highest Si concentration. The fact that the C:P ratio in roots was around 103% higher than in leaves (+ 1 and OL) is due to the significant increase in C concentration in the roots with a rise in Si dose and because they exhibited a lower P content than in leaves since they do not accumulate this element (Fig. 4i). Unlike the results obtained here, Neu et al.^23^ reported a decrease in the C:P ratio of wheat at all Si concentrations (0 a 50 g per pot).

As expected, C:Si was the stoichiometric ratio most influenced by Si doses, with reductions in all the plant parts assessed (Fig. 6j–l). The considerable decline in the C:Si ratio with rising Si doses is directly related to the lower C content and high Si uptake and accumulation capacity of the plant, evident in the linear increase observed in Si accumulation (Fig. 5d), in line with recent studies^9,31^.

Silicon accumulation was predominantly greater in leaves (+ 1 and OL) when compared to the stalk and roots, particularly the leaf tip (+ 1T and OLT). This high Si concentration gradient between the leaves and other plant parts is associated with Si metabolism in the plant, which involves uptake, radial transport in the root, xylem and intervascular transport, xylem unloading and deposit in the leaves^7^. In light of the above, our hypothesis (i) that C:N:P stoichiometry in sugarcane plants is modified by Si application was also confirmed.

### Outlook and perspectives

This is the first study to address the effect of Si concentrations on C:N:P stoichiometry in sugarcane plants. The findings obtained here indicate that Si alters C:N, C:P, N:P and C:Si differently according to the plant part. Thus, our results enabled a better understanding of the response of sugarcane plants to Si supply at cellular level based on chemical and physiological parameters, helping to optimise yield.

It is important to note that the use of pre-sprouted sugarcane seedlings (PSS) is growing, along with reports of problems related to early growth due to different stresses, especially water deficits and high temperatures at planting. This study indicated the biomass production of sugarcane plants obtained from mini-sets with low nutrient reserves increased linearly with Si application, due to more efficient photosynthesis and better heat tolerance. As such, this study provides valuable information for better initial establishment of PSS. However, long-term field studies are needed to assess yield and the residual effect of Si application.

Linear increases were also observed for C, N and P accumulation as well as biomass production, indicating that Si improved the N and P use efficiency, since the plant produced more biomass per unit of N or P absorbed, confirmed by the lower C:N and C:P ratios. This effect has also been reported in wheat plants^23^.

The greater accumulation of nutrients and C in biomass contributed to increasing stocks in the environment and improving C and nutrient cycling in the soil–plant-atmosphere system. Moreover, changes in stoichiometric ratios directly influenced the decomposition rate of plant residue. For example, lignin content was positively correlated with high C:N ratios, which decline as Si supply increases^26^. Thus, Si application in sugarcane plants enabled better C and nutrient cycling in the environment. The global cultivated area for sugarcane is approximately 27.4 million hectares^27^, highlighting the potential impact of this study in terms of yield and the environment.

## Methods

### Experimental setup

The experiment was conducted in a greenhouse (21°14′47.20″; 48°18′06.20″W), with daily relative humidity, maximum and minimum temperature readings (Fig. 7). A completely randomised design was used, with six repetitions and four silicon (Si) concentrations: 0, 0.8, 1.6 and 3.2 mM. The treatments were supplied continuously via a nutrient solution, using potassium silicate (128 and 104.6 g^-1^ of Si and K, respectively) as a Si source. Potassium content was balanced between the treatments using potassium chloride (KCl).

Plastic pots were filled with 3 dm^3^ of sand previously washed with water and 0.5 M HCl solution. One pre-sprouted sugarcane seedling (PSS) was transplanted into each pot at a depth of 5 cm. PSS is the process of germination of one node (mini-sets) containing a viable bud^38^. The sugarcane variety used was CTC4. Nutrient solution^39^ was applied to the pots on a daily basis until reaching about 80% water holding capacity, according to the methods described by Teixeira et al.^40^. The solution contained a modified iron source (Fe-EDDHMA) and pH adjusted to 5.5. Ionic strength of 10, 20, 30, 50 and 70% were applied 3, 8, 13, 18, 23 and 30 days after transplanting (DAT), respectively. In order to prevent salinization, the substrate of each pot was washed twice with 3 L of deionised water and pH adjusted to 5.5 every week.

### Photosynthetic parameters

At 90 DAT, photosynthetic parameters were assessed in the middle third of the leaf + 1 (first fully expanded leaf), avoiding the midrib. Gas exchange parameters were measured with a portable photosynthesis system (LcPro-SD, ADC BioScientificLtd., Hoddesdon, UK). Data were collected in the morning, between 9 and 11 a.m. Gas exchange measurements were taken at a constant light intensity of 1,800 µmol m^-2^ s^-1^ emitted by a blue-red LED light source, under natural CO_2_ conditions (403–428 ppm). Leaf temperature was kept at 30 ± 0.46ºC.

Net photosynthesis rate (A), leaf transpiration (E), stomatal conductance (gs) and intracellular CO_2_ concentration (Ci) were determined after stabilisation (3–5 min). Instantaneous carboxylation efficiency was calculated (*k* = A/Ci) and the maximum quantum efficiency of photosystem II (Fv/Fm) measured using a chlorophyll fluorometer (Opti-sciences®-Os30P+). Leaf disks were also collected to determine chlorophyll a, b, total chlorophyll (a + b) and carotenoid content, in accordance with the methodology described by Lichtenthaler^41^.

### Plant biomass

At 90 DAT, the shoots were cut horizontally and washed with deionised water, detergent solution (0.1% v/v), HCl solution (0.1% v/v) and then deionised water again. Next, they were stratified into stalks and leaves (+ 1 and other leaves – OL).The leaves were further stratified into three parts: sheath (S), tip (T) and base (B) of the leaf blade (without the central vein). In this article, these three leaf parts will be referred to as + 1T, + 1B and + 1S (first fully expanded leaf) and OLT, OLB and OLS (other leaves-OL). This leaf stratification method was chosen due to the distinct pattern of Si accumulation in this tissue.

Roots were also collected, washed with running water and then deionised water. The plant samples were dried in a forced-air oven at 65 °C until constant mass and the dry weight of each plant part was determined.

### Determination of C, N, P and Si

The dried plant matter was ground in a Wiley mill to determine carbon (C), nitrogen (N), phosphorous (P) and silicon (Si) concentrations. Carbon concentration was measured using the modified Walkley–Black method^42^, and P and N concentration according to the method described by Malavolta et al.^43^. Finally, Si concentration was extracted in line with Kraska and Breitenbeck^44^ and quantified using the method proposed by Korndörfer et al.^45^. Concentrations of C, N, P and Si were used to establish the C:N, C:P, N:P and C:Si ratios. The accumulation of C, N, P and Si was determined by multiplying the dry weight of each plant part by the respective concentrations of these elements.

### Statistical analyses

The data were submitted to analysis of variance at 5% probability and, when the F-test was significant, means were fit to the linear or quadratic model via regression analysis. The data were checked for outliers (Dixon’s Q test), normality (Shapiro–Wilk test) and homogeneity of variances (Levene’s test). Statistical analyses were performed in R 3.5.1 and graphs and regression analysis in SigmaPlot 10 (Systat Software, San Jose, CA).

## References

[1] Brackhage C, Schaller J, Bäucker E, Dudel EG (2013). Silicon availability affects the stoichiometry and content of calcium and micro nutrients in the leaves of common reed. Silicon.

[2] Ma, J. F., Miyake, Y. & Takahashi, E. Silicon as a beneficial element for crop plants. In *Silicon in Agriculture* (eds. Datnoff, L. E., Snyder, G. H. & Korndörfer, G, H.) 17–39 (Elsevier Science, Amsterdam, 2001)

[3] Savant NK, Korndörfer GH, Datnoff LE, Snyder GH (1999). Silicon nutrition and sugarcane production: A review. J. Plant Nutr..

[4] Brassioli FB, Prado RDM, Fernandes FM (2009). Avaliação agronômica da escória de siderurgia na cana-de-açúcar durante cinco ciclos de produção. Bragantia.

[5] McCray JM, Ji S (2012). Calibration of sugarcane response to calcium silicate on Florida histosols. J. Plant Nutr..

[6] de Camargo MS, Korndörfer GH, Wyler P (2014). Silicate fertilization of sugarcane cultivated in tropical soils. Field Crops Res..

[7] Yan GC, Nikolic M, Ye MJ, Xiao ZX, Liang YC (2018). Silicon acquisition and accumulation in plant and its significance for agriculture. J. Integr. Agric..

[8] Greger M, Landberg T, Vaculík M (2018). Silicon influences soil availability and accumulation of mineral nutrients in various plant species. Plants.

[9] Li Z (2018). Impacts of silicon on biogeochemical cycles of carbon and nutrients in croplands. J. Integr. Agric..

[10] Etesami H, Jeong BR (2018). Silicon (Si): Review and future prospects on the action mechanisms in alleviating biotic and abiotic stresses in plants. Ecotoxicol. Environ. Saf..

[11] Kim YH, Khan AL, Waqas M, Lee IJ (2017). Silicon regulates antioxidant activities of crop plants under abiotic-induced oxidative stress: A review. Front. Plant Sci..

[12] Epstein E (2009). Silicon: its manifold roles in plants. Ann. Appl. Biol..

[13] Maghsoudi K, Emam Y, Pessarakli M (2016). Effect of silicon on photosynthetic gas exchange, photosynthetic pigments, cell membrane stability and relative water content of different wheat cultivars under drought stress conditions. J. Plant Nutr..

[14] Wang J, Wen X, Zhang X, Li S, Zhang DY (2018). Co-regulation of photosynthetic capacity by nitrogen, phosphorus and magnesium in a subtropical Karst forest in China. Sci. Rep..

[15] Zhang G, Zhang L, Wen D (2018). Photosynthesis of subtropical forest species from different successional status in relation to foliar nutrients and phosphorus fractions. Sci. Rep..

[16] Reich PB, Schoettle AW (1988). Role of phosphorus and nitrogen in photosynthetic and whole plant carbon gain and nutrient use efficiency in eastern white pine. Oecologia.

[17] Veronica N (2017). Influence of low phosphorus concentration on leaf photosynthetic characteristics and antioxidant response of rice genotypes. Photosynthetica.

[18] Sitko K (2019). Influence of short-term macronutrient deprivation in maize on photosynthetic characteristics, transpiration and pigment content. Sci. Rep..

[19] Bassi D, Menossi M, Mattiello L (2018). Nitrogen supply influences photosynthesis establishment along the sugarcane leaf. Sci. Rep..

[20] Ma JF (2004). Role of silicon in enhancing the resistance of plants to biotic and abiotic stresses. Soil Sci. Plant Nutr..

[21] Verma KK (2019). The protective role of silicon in sugarcane under water stress: Photosynthesis and antioxidant enzymes. Biomed. J. Sci. Technol. Res..

[22] Ma JF, Takahashi E, Ma JF, Takahashi E (2002). Functions of silicon in plant growth. Soil, Fertilizer, and Plant Silicon Research in Japan.

[23] Neu S, Schaller J, Dudel EG (2017). Silicon availability modifies nutrient use efficiency and content, C:N: P stoichiometry, and productivity of winter wheat (*Triticum aestivum* L.). Sci. Rep..

[24] Raven JA (1983). The transport and function of silicon in plants. Biol. Ver..

[25] Deus ACF, Prado RM, Alvarez RCF, de Oliveira RLL, Felisberto G (2019). Role of silicon and salicylic acid in the mitigation of nitrogen deficiency stress in rice plants. Silicon.

[26] Klotzbücher T (2018). Variable silicon accumulation in plants affects terrestrial carbon cycling by controlling lignin synthesis. Glob. Chang. Biol..

[27] FAO. *Statistical Database*. https://www.fao.org/faostat/ (2019).

[28] Santos F, Diola V, Santos F, Borém A, Caldas C (2015). Physiology. Sugarcane.

[29] Ma D (2016). Silicon application alleviates drought stress in wheat through transcriptional regulation of multiple antioxidant defense pathways. J. Plant Growth Regul..

[30] Souza Junior JP, Prado RM, Sarah MMS, Felisberto G (2019). Silicon mitigates boron deficiency and toxicity in cotton cultivated in nutrient solution. J. Plant Nutr. Soil Sci..

[31] de Camargo MS (2019). Silicon fertilization improves physiological responses in sugarcane cultivars grown under water deficit. J. Soil Sci. Plant Nutr..

[32] Miyazawa Y, Yahata H (2006). Is the parameter electron transport rate useful as a predictor of photosynthetic carbon assimilation rate?. Bull. Inst. Trop. Agric. Kyushu Univ..

[33] Bokhtiar SM, Huang HR, Li YR, Dalvi VA (2012). Effects of silicon on yield contributing parameters and its accumulation in abaxial epidermis of sugarcane leaf blades using energy dispersive X-ray analysis. J. Plant Nutr..

[34] Mehrabanjoubani P, Abdolzadeh A, Sadeghipour HR, Aghdasi M (2015). Silicon affects transcellular and apoplastic uptake of some nutrients in plants. Pedosphere.

[35] Cooke J, Leishman MR (2012). Tradeoffs between foliar silicon and carbon-based defences: evidence from vegetation communities of contrasting soil types. Oikos.

[36] Schaller J, Brackhage C, Dudel EG (2012). Silicon availability changes structural carbon ratio and phenol content of grasses. Environ. Exp. Bot..

[37] Li Z (2018). Silicon enhancement of estimated plant biomass carbon accumulation under abiotic and biotic stresses. A meta-analysis. Agron. Sustain. Dev..

[38] Landell, M. G. A. *et al. Sistema de Multiplicação de Cana-de-açúcar com Uso de Mudas Pré-brotadas (MPB), Oriundas de Gemas Individualizadas* (Instituto Agronômico, 2012).

[39] Hoagland DR, Arnon DI (1950). The Water-culture Method for Growing Plants Without Soil.

[40] Teixeira GCM (2020). Silicon in pre-sprouted sugarcane seedlings mitigates the effects of water deficit after transplanting. J. Soil Sci. Plant Nutr..

[41] Lichtenthaler HK (1987). Chlorophylls and carotenoids: pigments of photosynthetic biomembranes. Methods Enzymol..

[42] Tedesco, M. J., Gianello, C., Bissani, C. A., Bohnen, H. & Volkweiss, S. J. *Análises de Solo, Plantas e Outros Materiais* (UFRGS, 1995).

[43] Malavolta, E., Vitti, G. C. & Oliveira, S. A. *Avaliação do Estado Nutricional das Plantas* (Potafos, 1997).

[44] Kraska JE, Breitenbeck GA (2010). Simple, robust method for quantifying silicon in plant tissue. Commun. Soil Sci. Plant Anal..

[45] Korndörfer, G. H., Pereira, H. S. & Nolla, A. *Análise de Silício no Solo, Planta e Fertilizante* (UFU, 2004).

